# Sexually Explicit Material and Its Relationship with Sociodemographic Variables, Sexual Satisfaction, and Relationship Satisfaction in a Spanish Sample

**DOI:** 10.3390/ijerph192114131

**Published:** 2022-10-29

**Authors:** Natalia Sánchez-Lamadrid, María del Mar Sánchez-Fuentes, Nieves Moyano, Reina Granados

**Affiliations:** 1 Department of Psychology and Sociology, Faculty of Human and Social Sciences, University of Zaragoza, 44003 Teruel, Spain; 2 Mind, Brain, and Behavior Research Center, University of Granada, 18011 Granada, Spain; 3 Department of Social Sciences, Faculty of Human and Social Sciences, University of La Costa, Barranquilla 080002, Colombia; 4 Faculty of Humanities and Science Education, University of Jaén, 23009 Jaén, Spain; 5 Department of Nursing, Faculty of Health Sciences, University of Granada, 18071 Granada, Spain

**Keywords:** sexually explicit material, pornography use, sexual satisfaction, relationship satisfaction

## Abstract

Previous research that has examined the use of sexually explicit material (SEM) in the Spanish population and its relationship with sociodemographic variables, sexual satisfaction, and satisfaction with the relationship are practically non-existent. Therefore, the main goal was to analyze the pattern of use of SEM (frequency, format, context, content, and purpose of its use) and its relationship with sociodemographic variables (sex, age, sexual orientation, educational level, religiosity, status of partner, number of sexual partners, and age of first exposure to SEM), sexual satisfaction, and relationship satisfaction. The sample consisted of 221 participants, with an average age equal to 29.88 years (*SD* = 9.73) and of Spanish nationality. All participants completed a sociodemographic questionnaire, as well as the Spanish adaptations of the Sexual Media Questionnaire, Global Measure of Sexual Satisfaction, and Global Measure of Relationship Satisfaction. Based on the results, it was found that men use SEM more frequently than women, that the age of first exposure is 14 years old, the most used pattern of SEM is internet websites with sexual content in which adult men and women appear, and the way to visualize it is alone as private stimulation. In addition, it was shown that a higher frequency of SEM use implies a decrease in sexual satisfaction and vice versa, while there is no association between the frequency of SEM use and satisfaction with the relationship. The importance of considering the use of SEM, both in research on sexual satisfaction and in clinical practice, is discussed.

## 1. Introduction

During the last decades, interest has been generated to understand the influence of media in the process of the development and exploration of the sexuality of individuals, mainly due to the increase in the availability of sexual content through the internet [1,2,3,4]. The “Triple A Engine” affirms that there are three factors for the internet to be a very powerful tool in relation to sexuality: accessibility, affordability, and anonymity. Thus, pornographic content is easily accessible to any user with internet access; most of it is free and you do not have to identify yourself with personal data to access it [5]. Previous research has concluded that numerous young internet users are exposed to pornographic content, most of them before they turn 18 [3], and this exposure is beginning at an increasingly younger age [5]. For this reason, most research on the use of pornography has focused on the adolescent or young population [3,4,6,7,8,9].

According to statistics from one of the largest pornography websites, Pornhub^®^, approximately 130 million people from all over the world access the Web, making it one of the most visited websites internationally. This represents about 36,000 million visits per year. Spain is ranked 11th out of the 20 countries that most view its page, with 38% of users being women and 62% men. The age group that makes the most visits is between 25 and 34 years old, followed by users aged between 18 and 24 [10,11]. In addition, during the global pandemic due to COVID-19, access to the Web increased by 61% in Spain [10].

On the other hand, it is worth noting the difference between eroticism and pornography. Although both seek to produce excitement, in eroticism, the sexual representation is more subtle or neutral, while in pornography, more violent or degrading sexual representations are included [12]. In the present study, sexually explicit material (SEM) will be analyzed, which includes both pornography and more subtle forms of sex (e.g., erotic literature).

The use of SEM has become an important source of information and/or “sexual education” for young people [13,14] that influences the processes of sexual socialization, which persist in adulthood [15]. This can lead to problems related to false expectations [4,12], objectification, degradation, and self-objectification [1,15]. Other variables related to the use of SEM, but less studied, are sexual satisfaction and satisfaction with the relationship. Although sexual satisfaction has been related to numerous variables [16,17], the relationship between sexual satisfaction and the use of SEM yields heterogeneous and even contradictory results. For example, although the research to date seems to agree that a higher frequency of SEM use is related to lower sexual satisfaction, some studies find this relationship in both men and women [9,18,19], others only in men [19,20,21], men with stronger religious beliefs [22], and in other research, there is no significant relationship between these two variables [8]. Similarly, the association between the use of SEM and satisfaction with the relationship does not seem to be clear either, finding both positive [18] and negative relationships; the latter only in men [19,21] and, mainly, in those who profess some religion [22].

In addition to the inconsistency in the results indicated, it is also important to mention that most studies have focused on men [21], heterosexual people [12,18,20,23,24], adolescents [8,25], and/or university students [3,7,9]. Another relevant aspect is that there are no previous studies in Spain that have examined the pattern of use of SEM, as well as its relationship with sociodemographic variables, sexual satisfaction, or satisfaction with the relationship. To the best of our knowledge, only Negy et al. [26] examined whether university students in the US and Spain considered using SEM to be equivalent to committing infidelity.

For all these reasons, this research aims to overcome some of the limitations exposed, since the pattern of use of SEM will be analyzed for the first time in the Spanish population in a sample made up of men and women of any sexual orientation, and the relationship between the use of SEM with sexual satisfaction and relationship satisfaction will be investigated. Therefore, the main objective of this study is to analyze the pattern of SEM use and its relationship with sociodemographic variables, sexual satisfaction, and satisfaction with the relationship. The specific objectives are: (1) to analyze the pattern of use of SEM (frequency, format, context, content, and purpose of its use); (2) to examine whether there are statistically significant differences in the pattern of SEM use based on sociodemographic characteristics (sex, age, sexual orientation, educational level, religiosity, partner status, number of sexual partners, and age of first exposure to SEM); and (3) to analyze whether there is a statistically significant relationship between the pattern of SEM use, sexual satisfaction, and partner satisfaction. The following hypotheses are proposed: (1) men will present a lower average than women at the age of first viewing SEM [3,6]; (2) men will use SEM more frequently than women [6,12]; and (3) people who have a higher frequency of SEM use will report lower levels of sexual satisfaction and partner satisfaction [12,19,20].

## 2. Materials and Methods

### 2.1. Participants

The sample was calculated using G*Power to perform goodness of fit tests (χ^2^), with a large effect size of 0.50, probability of alpha error of 0.05, and power (1 − β) of 0.95. The minimum sample size was 80, with an actual power of 0.95. We decided to expand the sample size a bit more to gain a better representation of several sociodemographic aspects that would be part of the analyses.

The sample consisted of 228 people; however, four of them were eliminated because they were minors (less than 18 years old); two participants were eliminated in order to maintain a homogeneous sample because their sex and gender did not match, that is, they were not cisgender; and one participant was eliminated since they only completed the information related to the sociodemographic questions. Thus, the final sample was made up of 221 participants (35.7% men and 64.3% women) with a mean age equal to 29.88 years (*SD* = 9.73). The mean age of first sexual intercourse was 17.33 (*SD* = 2.83), the mean number of sexual partners was 8.46 (*SD* = 9.26), and the mean age of first exposure to SEM was 14.3 years (*SD* = 3.46). The rest of the sociodemographic characteristics are presented in Table 1.

### 2.2. Instruments

Background questionnaire. This measure was used to collect information on sex, gender, age, educational level, professed religion, frequency of attendance at places of worship, frequency of religious practice other than attendance at places of worship, type of relationship, age of first sexual intercourse (oral, vaginal and/or anal), number of sexual partners, and nationality.

Kinsey Scale [27]. This scale assesses the most frequent sexual behavior using seven response options ranging from exclusively heterosexual to exclusively homosexual. In addition, we included an eighth option referring to asexuality.

Sexual Media Questionnaire [12]. This questionnaire is a self-report adapted to Spanish for the present study. The questionnaire is made up of six items related to the use of SEM, each with different response scales. Specifically, the self-report revealed the format, form, frequency, context, content, and purpose of use or viewing of explicit sexual material. The item “Age of the first-time viewing/using explicit sexual material” was added to the original questionnaire.

Global Measure of Sexual Satisfaction (GMSEX) [28]. We employed the Spanish version [29]. This measure assesses sexual satisfaction in the context of a relationship and is composed of five bipolar scales: very bad–very good; very unpleasant–very pleasant; very negative–very positive; very unsatisfying–very satisfying; worthless–very valuable. Each of these scales includes seven response alternatives, with scores ranging from 5 (*low sexual satisfaction*) to 35 (*high sexual satisfaction*), with higher scores indicating greater sexual satisfaction. In the present study, the instructions of the scale were modified in such a way that all the people, whether they were those who maintain a relationship, people with sporadic sexual partners, or single people, could answer the questions, responding based on the sexual relationships that they maintain with their current or past partner or with themselves. The Spanish version has adequate psychometric properties, with a Cronbach’s alpha of 0.92 [29]. In the present study, the internal consistency reliability was very good, with Cronbach’s alpha equal to 0.98.

Global Measure of Relationship Satisfaction (GMREL) [28]. We used the Spanish adaptation [29]. This measure assesses satisfaction with the relationship with an identical response format to that of the GMSEX. In the present study, only participants who reported being in a relationship (with or without cohabitation) completed this assessment. This scale presents a strong reliability, with a high internal consistency reflected with a Cronbach’s alpha of 0.94 [29]. In the present study, internal consistency reliability was also high, with Cronbach’s alpha equal to 0.98.

### 2.3. Procedure

We employed an incidental non-probabilistic procedure. The inclusion criteria were being 18 years old or older and of Spanish nationality. The participants answered an online survey. Previous research showed that online surveys allowed the recruitment of a higher diversity of participants, and this procedure was as reliable as traditional pencil and paper ones [30,31]. The survey was disseminated via a link that was distributed on social networks. The participants were informed of the study’s general aim, the characteristics of the scales, and what their participation implied. When the participant clicked on the link, the informed consent first appeared. Once the person had read it and accepted to participate in the study, questionnaires appeared. None of the questions were mandatory, except for the informed consent question. Participation was voluntary, anonymous, and participants received no compensation for their participation. The present research was approved by the Ethical Committee [blinded], whose opinion is based on the Declaration of Helsinki.

## 3. Results

### 3.1. Pattern of SEM Use

First, we employed frequency analysis to analyze the pattern of SEM use. Regarding the format of use of SEM, most participants (77.1%) reported that they used internet websites. Regarding the frequency of use, 30.8% of the participants reported that they never used SEM, followed by 17.6% who reported that they used it less than once a month, and only 3.2% more than once up to date. Most participants (93.4%) reported that they used SEM alone. Regarding the content, most participants (83.7%) indicated viewing sexual content between adult men and women, with only 3.9% of the participants reporting viewing degrading or violent acts. Finally, regarding the purpose, more than half of the participants (65.4%) reported using SEM as private stimulation, while only 5.1% used SEM to have sex with their partner (see Table 2).

### 3.2. Pattern of SEM Use Based on Sociodemographic Characteristics

Following the frequency analysis, to examine whether there were statistically significant differences in the pattern of SEM use based on sociodemographic characteristics, the chi-square test was performed. Statistically significant differences were found between the sociodemographic variable gender and the format of use of SEM (χ^2^ (5) = 20.53, *p* < 0.001), the frequency of use of SEM (χ^2^ (7) = 51.12, *p* < 0.001), and the purpose of using SEM (χ^2^ (9) = 18.56, *p* < 0.05). Regarding the age variable, there were only statistically significant differences with the purpose of using SEM (χ^2^ (279) = 330.83, *p* < 0.05). In the case of educational level, there were only statistically significant differences with the frequency of use of SEM (χ^2^ (35) = 50.27, *p* < 0.05). On the other hand, sexual orientation showed statistically significant differences with the content of the SEM used (χ^2^ (30) = 160.71, *p* < 0.001). With the professed religion, there were statistically significant differences with the content of the SEM used (χ^2^ (24) = 52.27, *p* < 0.001). Regarding the frequency of attendance at places of worship, statistically significant differences were found with the purpose of using SEM (χ^2^ (36) = 54.44, *p* < 0.05) and between the frequency of practicing religion other than attendance at places of worship and the format of use (χ^2^ (25) = 45.40, *p* < 0.05) and the content of the SEM used (χ^2^ (30) = 68.29, *p* < 0.001). Finally, there were statistically significant differences between the number of sexual partners and the content of the SEM used (χ^2^ (156) = 255.35, *p* < 0.001). The rest of the sociodemographic variables and those related to the use of SEM did not show statistically significant differences (see Table 3).

Next, we examined, by means of the chi-square test, if there was a statistically significant relationship between the pattern of use, specifically, format, context, content, and purpose of use of SEM with sexual satisfaction and satisfaction with the relationship. In addition, Pearson’s correlation analysis examined the relationship between frequency of SEM use with sexual satisfaction and relationship satisfaction. First, it was found that there were statistically significant differences between SEM format and sexual satisfaction (χ^2^ (110) = 136.19, *p* < 0.05) and relationship satisfaction (χ^2^ (80) = 121.30, *p* < 0.05), as well as between the purpose of using SEM and sexual satisfaction (χ^2^ (198) = 275.44, *p* < 0.001). Second, it was found that there was a significant relationship between the frequency of SEM use and sexual satisfaction (*r* = −0.17, *p* < 0.05).

Finally, we analyzed whether there were statistically significant differences in the average age of first viewing and frequency of use of SEM between men and women. Regarding the average age of the first SEM viewing, there were statistically significant differences (*U* = 3394.00, *p* < 0.001), with the average age of men being 13.16 years (*SD* = 3.50) and that of women equal to 15.01 years (*SD* = 0.48). Regarding the frequency of use, there were also statistically significant differences between men and women (χ^2^ (7) = 87.35, *p* < 0.001), with the frequency of use being higher in men (average range = 159.68) than in women (mean range = 84.80).

## 4. Discussion

Given the lack of studies that jointly explore the relationship between the pattern of SEM use, sexual satisfaction, satisfaction with the relationship, and sociodemographic characteristics, the main goal of this work was to analyze the use of SEM in the Spanish population and to understand how it relates to the variables mentioned. The results show that men and women differ in terms of the format, frequency, content, and purpose of using SEM. It should be noted that men reported a lower age of exposure to SEM and a higher frequency of its use than women. Regarding the format, frequency, and context, differences were also found depending on the frequency of religious practice other than attendance at places of worship, the level of studies, and the status of a couple, respectively. We also verified that there were differences between the content of the SEM used according to sexual orientation, professed religion, and the frequency of religious practice other than attendance at places of worship. Age, couple status, and frequency of attendance at places of worship showed differences with respect to the purpose for which the SEM was used. There was no significant relationship between the frequency of SEM use and satisfaction with the relationship, although it was observed that a greater frequency of SEM use was related to less sexual satisfaction.

Regarding the pattern of use of SEM, we verified that the average age of the first exposure to SEM was 14.3 years, a similar result to previous studies [32], and the first exposure coincided with puberty, a vital stage where sexual development and exploration begins [2]. The most widely used format was that of websites on the internet, a result that is not unusual due to affordability, ease of access, and anonymity [5]. The most used context was “alone” and only 6.6% indicated that they viewed SEM with their partner. Previous studies have also concluded that most people use SEM alone [32,33], which could be explained by taking into account that on most occasions, it is used as stimulation for the masturbatory practice that is usually carried out most frequently without the presence of the partner [21,34]. In fact, in this research, the participants indicated that the main purpose of use was “as private stimulation”, followed by “helping me fantasize” or “relaxing”. Regarding the content, the most frequent was that of heterosexual sexual acts and the least used was that related to degrading and/or violent acts, which can be explained, first, because the majority of the participants identified themselves as heterosexual, and second, because most of the sample was made up of women and they tend to reject this type of material, preferring the neutral and subtle material known as erotic [12]. In relation to this aspect, we verified that the highest frequency of use of SEM, followed by those who do not use SEM, was less than once a month, which could also be explained by the majority gender of the sample of the present study. In this sense, previous research has shown that women use SEM less frequently than men [6,12].

Regarding the existence of differences based on certain sociodemographic variables and the pattern of use of SEM, it can be stated that according to sex, a specific SEM format, frequency, and purpose are used. Men use the virtual format (web pages) more while women prefer the literary format, which may be due to their erotic preference [12]. According to the proposed hypothesis, and coinciding with previous studies [2,6,12,20,32], men use SEM more frequently than women. This result may probably be due to traditional gender roles [35] and sexual double standards [36,37] that are related to more restrictive sexual behavior in women. Men also use SEM at an earlier age [2,6,12,20,32], more frequently, and with the aim of reducing stress or boredom compared to women. Women more commonly use SEM out of curiosity, to fantasize, and as a method of sexual education. However, the purpose also varies according to age, that is, at older ages, the use of SEM was mostly used in private stimulation [4]. Regarding the level of education, there were only differences in the frequency of use, with people with a lower level of education being the ones who used SEM more frequently. Previous research that has examined the level of education and the use of SEM is practically non-existent, so it is recommended to carry out future research that helps to understand the relationship between these variables. There were also differences in the content based on sexual orientation. As expected, homosexual people preferred homosexual content, thus coinciding with their sexual preferences. Regarding partner status, there were differences in the context and purpose of using SEM. These results are similar to those found by Hald and Malamuth [32], where only people with a stable relationship visualized SEM with their partner; this is not the case with people with occasional sexual partners. Finally, differences were also found in the pattern of use of SEM and religion. In this regard, for participants who professed a religion and practiced it more often (in this case, from “a few times a week” to “daily”), the content became exclusively heterosexual. In addition, the purpose of using SEM in people with a higher frequency of attendance at places of worship was reduced exclusively to private stimulation. This may be due to a possible cognitive dissonance since the action of using SEM is contradictory to religious beliefs, and although this paradox can be manifested at a psychological level, it does not seem to influence what is related to sexuality [22]. Therefore, the feelings of guilt of religious people do not seem to be linked to the use of SEM and their ability to enjoy sex [38]. In this way, these people do use SEM, but adjust the content and purpose to what they believe is morally “more acceptable” in order to reduce, as far as possible, the cognitive dissonance that is generated.

Regarding the pattern of use of SEM and sexual satisfaction, a relationship with frequency was found. In accordance with the proposed hypothesis, and in line with previous studies [9,19,20,22,39], we found that a greater frequency of SEM use is related to less sexual satisfaction. This inverse relationship may be because SEM can act as a primary source for sexual arousal, and its frequent use can generate high expectations regarding sexual relationships that may not coincide with reality [4,12]. In addition, considering the Interpersonal Exchange Model of Sexual Satisfaction, if expectations are not met, people reported lower sexual satisfaction [40,41]. The purpose of using SEM can also influence sexual satisfaction or vice versa [4,20,23]. In addition, in the present work, differences were observed in the format of SEM use and sexual satisfaction and satisfaction with the relationship, which may be since, depending on the format used, the content may be pornographic or erotic. However, there was no relationship between frequency of SEM use and relationship satisfaction, possibly because relationship satisfaction is associated more with quality and partner communication skills [23].

The present study is not without limitations. First, we employed an intentional non-probabilistic sampling, so the results cannot be generalized to the general Spanish population. In addition, the sample was made up mostly of women and heterosexual people, so it is recommended that more heterogeneous samples be used in future research. Likewise, future studies are needed to analyze whether the pattern of SEM use predicts sexual satisfaction or, on the contrary, the level of sexual satisfaction predicts the pattern of SEM use.

## 5. Conclusions

This research shows for the first time the pattern of use of SEM (i.e., frequency, format, context, content, and purpose of its use) in a Spanish sample, as well as its relationship with sociodemographic variables, sexual satisfaction, and satisfaction with the relationship. In general terms, it was found that men use this material at an earlier age and more frequently than women. In addition, there are differences between men and women in the format, content, and purpose of use of SEM. Likewise, a higher frequency of SEM use is related to lower sexual satisfaction, possibly due to the creation of false expectations with sexual relations in real contexts, although its use does not seem to influence satisfaction with the relationship. Future research is necessary to understand if the pattern of SEM use predicts sexual satisfaction or vice versa, with the ultimate purpose of designing and implementing sexual education programs, as well as the consideration of the pattern of SEM use in clinical practice.

## Figures and Tables

**Table 1:** Sociodemographic characteristics.

Variables	*n*	%
Educational level		
Primary education	5	2.33
Secondary education	6	2.77
High school	32	14.5
Training cycle (intermediate grade, higher, or education in regulated arts)	53	24
University	90	40.7
Postgraduate	35	15.8
Sexual orientation		
Heterosexual	178	80.5
Heterosexual with sporadic homosexual contact	9	4.1
Bisexual	21	9.5
Homosexual with sporadic heterosexual contact	3	1.4
Homosexual	9	4.1
Asexual	1	0.5
Couple status		
Single without sexual partners	44	19.9
Single with occasional sexual partners	35	15.8
Couple relationship without cohabitation	62	28.1
Couple relationship with cohabitation	80	36.2
Professed religion		
None	126	57
Catholic	87	39.4
Islamic	1	0.5
Buddhist	2	0.9
Other	5	2.3
Frequency of attendance at places of worship		
Never	124	56.1
Once a year	28	12.7
A few times a year	60	27.1
A few times a month	3	1.4
A few times a week	6	2.7
Frequency of religious practice, other than attendance at places of worship		
Never	144	65.5
Once a year	9	4.1
A few times a year	36	16.4
A few times a month	10	4.5
A few times a week	10	4.5
Daily	11	5

**Table 2:** Pattern of SEM use.

Variables	*n*	%
SEM usage format		
Internet websites (online)	121	77.1
Literature (no images)	20	12.7
Magazines, drawings, or images	5	3.2
Videos or movies (offline)	5	3.2
Chat or cybersex	4	2.5
Other	2	1.3
Frequency of use of SEM		
Never	68	30.8
Less than once a month	39	17.6
Between 1 and 2 times a month	28	12.7
Between 3 and 4 times a month	22	10.0
Between 1 and 2 times a week	25	11.3
Between 3 and 4 times a week	16	7.2
Between 5 and 6 times a week	8	3.6
Once a day	8	3.6
More than once a day	7	3.2
SEM context of use		
Alone	141	93.4
In a couple	10	6.6
Employed SEM content		
Women masturbating alone	4	2.6
Men masturbating alone	1	0.7
Sexual acts between adult women and men	128	83.7
Sexual acts between adult men	6	3.9
Sexual acts between adult women	6	3.9
Violent or degrading sexual acts	6	3.9
Others	2	1.3
Purpose of use of SEM		
Private stimulation	102	65.4
Sex with my partner	8	5.1
Fantasize	12	7.7
Reduce stress	9	5.8
Boredom	4	2.6
Curiosity	7	4.5
As a method of sex education	6	3.8
My partner does not want to be as intimate as I would like	2	1.3
My partner’s sexual problems	2	1.3
Availability of my partner	4	2.6

**Table 3:** Pattern of SEM use based on sociodemographic characteristics.

Sociodemographic Variables	SEM Usage Pattern Variables	*χ* ^2^	*gl*	*p*
Sex	Format	20.53	5	**
	Frequency	51.12	7	**
	Context	-	-	0.06
	Contents	19.50	6	*
	Purpose	18.56	9	*
Age	Format	-	-	0.69
	Frequency	-	-	0.62
	Context	-	-	0.92
	Contents	-	-	1
	Purpose	50.27	35	*
Educational level	Format	-	-	0.34
	Frequency	50.27	35	*
	Context	-	-	0.83
	Contents	-	-	0.12
	Purpose	-	-	0.57
Sexual orientation	Format	-	-	0.92
	Frequency	-	-	0.88
	Context	-	-	0.46
	Contents	160.71	30	**
	Purpose	-	-	0.45
Couple status	Format	-	-	0.65
	Frequency	-	-	0.21
	Context	17.55	3	**
	Contents	-	-	0.17
	Purpose	43.22	27	*
Professed religion	Format	-	-	1
	Frequency	-	-	0.98
	Context	-	-	0.96
	Contents	52.27	24	**
	Purpose	-	-	0.62
Frequency of attendance at places of worship	Format	-	-	0.97
	Frequency	-	-	0.23
	Context	-	-	0.97
	Contents	-	-	0.07
	Purpose	54.44	36	*
Frequency of religious practice other than attendance at places of worship	Format	45.40	25	*
	Frequency	-	-	0.22
	Context	-	-	0.64
	Contents	68.29	30	**
	Purpose	-	-	0.37
Number of sexual partners	Format	-	-	0.27
	Frequency	-	-	0.71
	Context	-	-	0.83
	Contents	-	-	0.76
	Purpose	-	-	0.98
Age of first sexual activity	Format	-	-	0.21
	Frequency	-	-	0.72
	Context	-	-	0.90
	Contents	-	-	0.92
	Purpose	-	-	0.13
Age of first viewing of SEM	Format	-	-	0.83
	Frequency	-	-	0.23
	Context	-	-	0.44
	Contents	-	-	0.88
	Purpose	-	-	0.36

Note. ** *p* < 0.01; * *p* < 0.05.

## Data Availability

The data presented in the study are available o request from the corresponding author. The data are not publicly available due to privacy.
